# Controlled Heterotypic Pseudo-Islet Assembly of Human β-Cells and Human Umbilical Vein Endothelial Cells Using Magnetic Levitation

**DOI:** 10.1089/ten.tea.2019.0158

**Published:** 2020-04-16

**Authors:** Max Urbanczyk, Aline Zbinden, Shannon L. Layland, Garry Duffy, Katja Schenke-Layland

**Affiliations:** ^1^ Department of Women's Health, Research Institute for Women's Health, Eberhard Karls University Tübingen, Tübingen, Germany.; ^2^ Department of Anatomy, School of Medicine, College of Medicine, Nursing and Health Sciences, National University of Ireland Galway, Galway, Ireland.; ^3^ The Natural and Medical Sciences Institute (NMI) at the University of Tübingen, Reutlingen, Germany.; ^4^ Cluster of Excellence iFIT (EXC 2180) “Image-Guided and Functionally Instructed Tumor Therapies,” Eberhard Karls University Tübingen, Tübingen, Germany.; ^5^ Department of Medicine/Cardiology, Cardiovascular Research Laboratories, University of California, Los Angeles, California.

**Keywords:** diabetes, islets, β-cells, insulin, endothelial cells, 3D

## Abstract

β-Cell functionality and survival are highly dependent on the cells' microenvironment and cell–cell interactions. Since the pancreas is a highly vascularized organ, the crosstalk between β-cells and endothelial cells (ECs) is vital to ensure proper function. To understand the interaction of pancreatic β-cells with vascular ECs, we sought to investigate the impact of the spatial distribution on the interaction of human cell line-based β-cells (EndoC-βH3) and human umbilical vein endothelial cells (HUVECs). We focused on the evaluation of three major spatial distributions, which can be found within human islets *in vivo*, in tissue-engineered heterotypic cell spheroids, so-called pseudo-islets, by controlling the aggregation process using magnetic levitation. We report that heterotypic spheroids formed by spontaneous aggregation cannot be maintained in culture due to HUVEC disassembly over time. In contrast, magnetic levitation allows the formation of stable heterotypic spheroids with defined spatial distribution and significantly facilitated HUVEC integration. To the best of our knowledge, this is the first study that introduces a human-only cell line-based *in vitro* test system composed of a coculture of β-cells and ECs with a successful stimulation of β-cell secretory function monitored by a glucose-stimulated insulin secretion assays. In addition, we systematically investigate the impact of the spatial distribution on cocultures of human β-cells and ECs, showing that the architecture of pseudo-islets significantly affects β-cell functionality.

**Impact statement:**

Tissue engineering of coculture systems containing β-cells and endothelial cells (ECs) is a promising technique to stimulate β-cell functionality. In this study, we analyzed human pancreatic islet tissue and revealed three different native distributions of β-cells and ECs. We successfully recreated these distributions *in vitro* by employing magnetic levitation of human β-cells and ECs, forming controlled heterotypic pseudo-islets, which enabled us to identify a significant impact of the pseudo-islet architecture on insulin secretion.

## Introduction

In 2018, 425 million people were known to suffer from diabetes mellitus (DM), with millions more remaining undiagnosed.^1,2^ The number of DM patients is estimated to increase to >600 million by 2045.^1^ DM is a chronic disease, where the physiological feedback loop of blood glucose regulation is impaired by either the reduction of β-cell mass and insulin production, type 1 DM (T1D), or by a defective response to insulin in tissues, type 2 DM.^3^ The T1D incidence rate increases annually by 3% worldwide, similarly increasing the number of patients with various complications accompanied by T1D such as cardiovascular diseases, peripheral vascular diseases, or nephropathy. T1D patients rely on life-long medication and treatment, such as exogeneous insulin replacement or, in more severe cases, immunosuppression after pancreatic β-cell transplantation.^4,5,6^ Attempts toward the long-term treatment of T1D by β-cell transplantation frequently fails due to an impaired graft survival as a result of lost extracellular matrix (ECM)^7,8^ or lack of vascularization accompanied by hypoxia after islet isolation.^4,9,10^

The pancreas is a highly vascularized organ,^11^ which is particularly true for the β-cell-containing islets of Langerhans, with a capillary density of 400 capillaries/mm^2^.^12^ During homeostasis, endothelial cells (ECs) within the pancreas are vital for the maintenance of the microenvironment of the insulin-producing β-cells by upregulation of ECM-associated genes^13^ to secrete crucial basement membrane proteins.^14–17^ β-cells and ECs share a natural proximity and are, by necessity, exposed to each other and each other's products.^9,18^ The combination of pancreatic β-cells and ECs might be the key to stimulate β-cell survival and functionality and help to overcome the high transplant loss rate of ∼60%.^5,19^ However, understanding the interactions between β-cells and ECs is required to optimize those transplantable grafts.^19^ Unfortunately, the availability of islets of Langerhans explants is limited.^20^ Therefore, scientists rely on cell lines as an alternative model to mimic the functionality of islets of Langerhans *in vitro*. Current research on T1D focuses on the understanding of β-cell re-establishment by the investigation of complex cellular organoids, such as heterotypic cell aggregates from β-cells and ECs,^9,18^ so-called pseudo-islets. Bioengineered prevascularized grafts combining β-cells and ECs *ex vivo* have been reported,^21–24^ potentially reducing the required time to develop angiogenesis and revascularization *in vivo*, facilitating graft survival after transplantation.^13,25^ Apart from the aspect of increased survival,^22^ distinct groups have shown that β-cell and EC cocultures impact graft vascularization and increase β-cell functionality.^9,10,14,18,26^ However, to the best of our knowledge, functional stimulation in cell line-based β-cell systems has only been shown in intraspecific cocultures of rodent cells^14^ and interspecific cocultures of rodent β-cells and human ECs^18,26,27^ *in vitro*. A human-based coculture *in vitro* test system using ECs that stimulate β-cell functionality has not been presented, since only rodent β-cell lines (e.g., INS-1E or MIN6) were shown to be glucose responsive, whereas the functionality of human β-cell lines was controversially discussed.^28^ However, evaluation of rodent pancreatic islets revealed major differences in the spatial cell and ECM distribution^29^ and in insulin secretion mechanisms,^30^ emphasizing the need for a human cell line-based model.

**FIG. 1.**
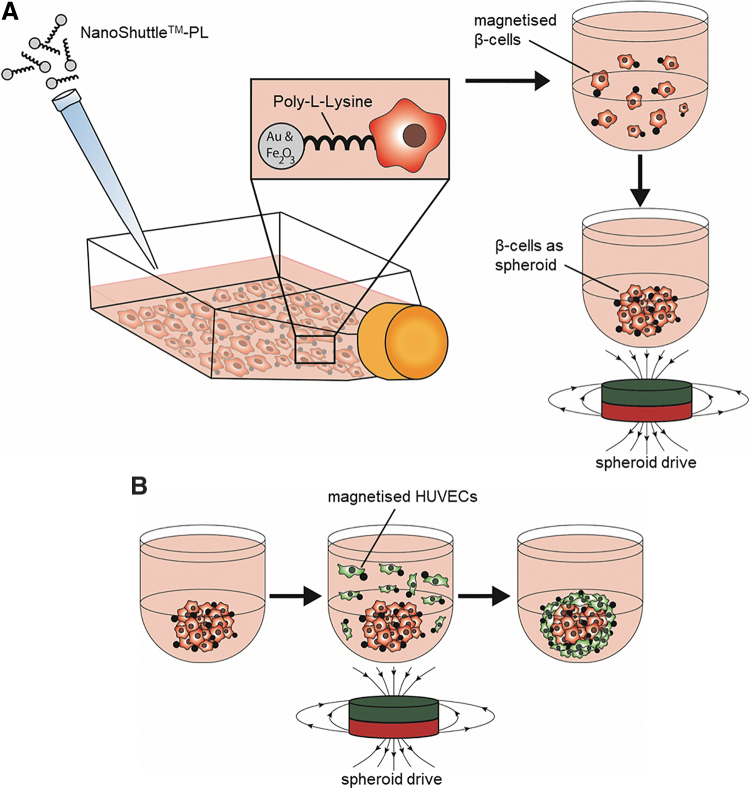
Schematic of magnetic levitation to create two-layered heterotypic spheroids. **(A)** NanoShuttle™-PL is added to a T25 flask containing cells and incubated at 37°C overnight. After detaching, β-cells are added into a low adherence u-bottom 96-well plate and aggregated by applying external magnetic forces using the spheroid drive. **(B)** HUVECs are treated with NanoShuttle-PL overnight, and are added as single cell suspension to the already formed spheroids from step A. Applying a magnetic field through the spheroid drive forces the HUVECs to aggregate around the preformed β-cell-containing spheroids creating a two-layered pseudo-islet composed of β-cells and HUVECs. HUVECs, human umbilical vein endothelial cells.

Recently, the conditionally immortalized, nonproliferating and glucose-sensitive human β-cell line EndoC-βH3 was developed, enabling research on β-cells with a human genetic background without the need to use donor pancreas explants.^31,32^ In this study, we investigated whether the spatial distribution of the EndoC-βH3 cells and human umbilical vein endothelial cells (HUVECs) has an impact on the insulin production within three-dimensional (3D) pseudo-islet cultures. The heterotypic spheroids were formed by spontaneous or controlled aggregation using magnetic levitation.^33^ The procedure of magnetic levitation functionalizes cells on their surfaces using a combination of poly-l-lysine with gold and iron oxide nanoparticles. Afterward, cellular movement and aggregation can be guided using external magnetic fields (Fig. 1), enabling a controlled aggregation to form (multi-) cellular 3D spheroids with defined spatial distributions.^19^

## Materials and Methods

### Cell culture

If not stated otherwise, all cell types used in this study were cultured under standard humidified cell culture conditions (37°C, 5% carbon dioxide).

EndoC-βH3 cells (Univercell Biosolutions, Paris, France), a conditionally immortalized human pancreatic β-cell line, was cultured according to the manufacturer's instructions. In brief, cells were cultured in coated (βcoat^®^; Univercell Biosolutions) T25 flasks at a density of 70,000 cells/cm^2^ in culture medium (OPTIβ1^®^; Univercell Biosolutions) supplemented with 10 μg/mL puromycin (ant-pr; InvivoGen, San Diego, CA) and passaged every 7 days. The immortalizing transgenes were removed by a 21-day treatment with 4-hydroxy tamoxifen (H7904; Sigma-Aldrich, Schnelldorf, Germany) to obtain nonproliferative β-cells that closely mimic human β-cells (Supplementary Fig. S1).

Vascular endothelial growth factor prescreened HUVECs (C-12205; PromoCell, Heidelberg, Germany) were cultured in EC growth medium (C-22010; PromoCell) in T25 flasks. Cells were passaged at a density of 80–90%. Medium was changed every 2–3 days. HUVECs were used between passages 2 and 6.

Rat insulinoma INS-1E cells (a kind gift of P. Maechler from the University of Geneva) were cultured in adjusted RPMI 1640 (12633012; Gibco, Thermo Fisher Scientific, Darmstadt, Germany) containing 10 mM HEPES (Gibco), 50 μM 2-mercapto-ethanol (Sigma-Aldrich), 1 mM pyruvate (Gibco), 5% fetal bovine serum (Sigma-Aldrich), 100 iU/mL penicillin, and 100 μg/mL streptomycin. The medium was changed every 2–3 days. The cells were passaged or seeded at a confluency of 80–90%.

### Pseudo-islet assembly

**FIG. 2.**
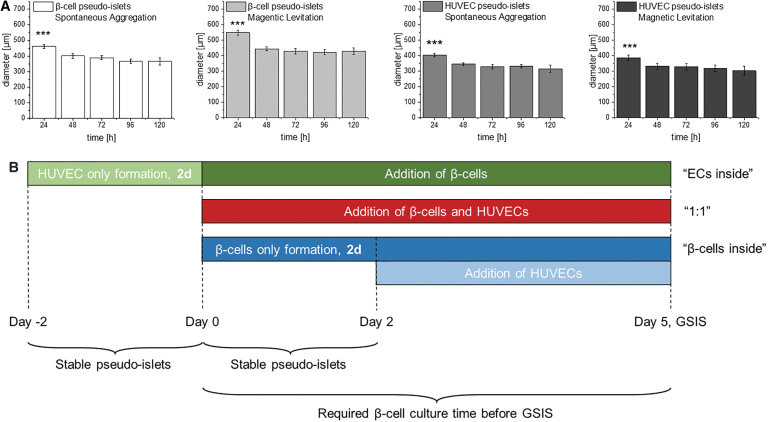
Illustration of experimental timeline with regard to external constraints. **(A)** Pseudo-islets require 48 h of culture time to form stable aggregates independent from the culture method or cell type. **(B)** Experimental timeline required to form stable pseudo-islets, and β-cells are cultured for 5 days according to manufacturer's protocol before GSIS testing is performed. One-way ANOVA, *n* = 10, ***p < 0.001. ANOVA, analysis of variance; ECs, endothelial cells; GSIS, glucose-stimulated insulin secretion.

For a controlled aggregation of cells within pseudo-islets, magnetic levitation was employed using the 96-well Bioprinting Kit (655840; Greiner Bio-One, Frickenhausen, Germany). In detail, β-cells and HUVECs were treated overnight with NanoShuttle™-PL at a concentration of 40 μL/mL in media according to the manufacturer's protocol. After the NanoShuttle-PL treatment, magnetized β-cells and HUVECs, as well as conventionally cultured β-cells and HUVECs were individually detached from their flasks using 0.25% Trypsin/EDTA. For coculture experiments, all cells were seeded at a density of 5000 cells/50 μL in corresponding cell culture media per well in a low adherence u-bottom 96-well plate (650970; Greiner Bio-One). We used three different conditions: (1) “1:1,” where the same amount of HUVECs and β-cells were mixed before seeding to form pseudo-islets with a heterogeneous distribution of both cell types; (2) “ECs inside,” where HUVECs were surrounded by β-cells; and (3) “β-cells inside,” where β-cells were surrounded by HUVECs (Fig. 1). In detail, for the “1:1” condition, magnetized or conventionally cultured cells of both cell types were seeded for magnetic levitation or spontaneous aggregation at day 0, placed atop the spheroid drive (Greiner Bio-One) for 1 h for exposure to an external magnetic field, followed by culture under standard conditions for 5 days. For the “ECs inside” condition, magnetized HUVECs for magnetic levitation or standard HUVECs for spontaneous aggregation were seeded at day 0 and placed atop the spheroid drive for 1 h, followed by culture under standard conditions. At day 2 of the culture, magnetized β-cells or standard β-cells were added to the HUVEC spheroids formed by either magnetic levitation or spontaneous aggregation, placed atop the spheroid drive for 1 h, followed by culture under standard conditions for 5 days. For the “β-cells inside” condition, magnetized β-cells for magnetic levitation or standard β-cells for spontaneous aggregation were seeded at day 0, placed atop the spheroid drive for 1 h, followed by culture under standard conditions. After 2 days of culture, magnetized HUVECs or standard HUVECs were added to the β-cell spheroids formed by magnetic levitation or spontaneous aggregation, placed atop the spheroid drive for 1 h, followed by culture under standard conditions for additional 3 days. A graphical representation of the experimental timeline can be found in Figure 2. Spontaneous aggregation was not guided by the spheroid drive assembly. The cell ratio within the pseudo-islets was 5000 β-cells to 5000 HUVECs, and the cell culture medium was a 1:1 mixture of OPTIβ1 and EC growth medium throughout all experiments.

### Terminal deoxynucleotidyl transferase-mediated nick-end labeling assay

Cell death analysis was performed using pseudo-islets composed of either magnetized β-cells and HUVECs or conventionally cultured β-cells. After culture, the pseudo-islets were fixed with 4% paraformaldehyde (Sigma-Aldrich) overnight at 4°C, embedded in paraffin, and cut into 3 μm thick sections. Terminal deoxynucleotidyl transferase-mediated nick-end labeling (TUNEL) staining was performed using the Click-iT TUNEL kit (C10245; Thermo Fisher Scientific). In brief, sections were deparaffinized in xylene and rehydrated through graded ethanol (100–50%) and vascular endothelial (VE)-water washes, and permeabilized using 0.25% Triton-X for 20 min. Terminal deoxynucleotidyl transferase reaction buffer was applied for 1 h at 60°C before slides were incubated with Click-iT reaction buffer for 30 min. Counterstaining was performed using a 4′,6-diamidino-2-phenylindole (DAPI; Sigma-Aldrich) solution with a concentration of 2 μg/mL for 10 min before mounting with ProLong^®^ Gold antifade mounting medium (P36934; Invitrogen™, Darmstadt, Germany).

### Glucose-stimulated insulin secretion assay

Before performing the glucose-stimulated insulin secretion (GSIS) assays, all pseudo-islets were cultured in starvation medium (OPTIβ2^®^; Univercell Biosolutions) for 24 h under standard conditions. Afterward, the pseudo-islets were washed twice with β-Krebs medium, consisting of KREBS-buffer (βKREBS^®^; Univercell Biosolutions) containing 10% bovine serum albumin (A-9576; Sigma-Aldrich), followed by an incubation for 1 h in β-Krebs medium for synchronization as previously described.^31^ Subsequently, the pseudo-islets were alternatingly incubated in basal β-Krebs medium (Krebs 1), β-Krebs medium with 20 mmol/L d-glucose (20 mM glucose) (Gibco), and basal β-Krebs medium (Krebs 2) for 1 h under each condition. After each incubation, the supernatant was removed and stored at −20°C until further analysis. The insulin content of all supernatants was analyzed using an ultrasensitive human insulin enzyme-linked immunosorbent assay (10-1132-01; Mercodia, Uppsala, Sweden). Insulin content was normalized by number of pseudo-islets.

### GSIS index

The GSIS index from Krebs 1 to 20 mM glucose was calculated using the formula: (insulin secretion of 20 mM glucose)/(insulin secretion of Krebs 1); the GSIS index from 20 mM glucose to Krebs 2 was calculated accordingly using the formula: (Insulin secretion of Krebs 2)/(insulin secretion of 20 mM glucose). The standard deviation was calculated using the formula for propagation of error.

### Immunofluorescence staining and microscopy

Pseudo-islets were processed to 3 μm thick sections and deparaffinized as described earlier. Adult human pancreatic tissue sections were purchased from Novus Biologicals (NBP2-30191; Novus Biologicals, Bio-Techne GmbH, Wiesbaden, Germany). For all sections, heat-mediated antigen retrieval in TRIS-EDTA (pH = 9.0) and citrate (pH = 6.0) buffer was performed. All sections were blocked in 2% goat block serum. For immunofluorescence (IF) staining, the following primary antibodies and dilutions were used: platelet endothelial cell adhesion molecule-1 (CD31; sc-71872; Santa Cruz, USA, 1:50), insulin (A0564; DAKO, Frankfurt, Germany, 1:200), epithelial cadherin (E-cadherin) (ab76055; Abcam, 1:250), Ki67 (ab15880; Abcam, 1:1000). For IF staining of CD31 and insulin, the sections were pretreated with 0.1% Triton-X 100 for 10 min. The primary antibodies were incubated overnight at 4°C. The following secondary antibodies were used at a dilution of 1:250: Alexa Fluor^®^ goat anti-mouse immunoglobulin G (IgG) 1 (488), goat anti-mouse IgG 2a (488) and goat anti-guinea pig (594) (all Invitrogen). Isotype controls were included. Counterstaining was performed using DAPI solution (Sigma-Aldrich) with a concentration of 2 μg/mL for 10 min before mounting with ProLong Gold antifade mounting medium (Thermo Fisher Scientific). IF staining and bright field (BF) images from pseudo-islets were captured at 10 × or 20 × magnification and 1.0 × tube lens, IF staining images from pancreatic tissues were captured at 63 × magnification and 1.0 × tube lens using an Observer Z1 light microscope from Zeiss (Carl Zeiss, Jena, Germany). BF images of long-term stability were obtained with 10 × magnification and 1.6 × tube lens.

### Image analysis

Blinded TUNEL images were quantified by two unbiased observers. Cells were identified as TUNEL^+^ when exhibiting a clear green and blue double staining. The cell death ratio was calculated by quantifying TUNEL^+^ cells, normalized to the DAPI count per pseudo-islet using the formula: normalized cell death ratio = (TUNEL^+^ cells in pseudo-islet)/(DAPI count per pseudo-islet).

CD31 expression per insulin was quantified using IF images. Regions of interest (ROIs) were drawn focusing on the insulin staining, which was then overlaid onto the CD31 staining. Grey value intensities (GVI) were measured for both insulin and CD31. The CD31 ratio was calculated using the formula: ratio = (GVI of CD31 in ROI)/(GVI of insulin in ROI).

E-cadherin expression was measured by GVI analysis of DAPI^+^ regions within pseudo-islets. All measurements were performed using ImageJ.

To assess the EC location within pseudo-islets of the “β-cells inside” distribution formed by magnetic levitation, the pseudo-islets were divided in two areas with the same size: A_inside_ and A_periphery_, where A_inside_ had a radius of 0.707 days. The GVI of CD31 staining was measured in A_inside_ and multiplied by the area of A_inside_, to obtain the total CD31 expression within the corresponding area. The same procedure was executed for A_periphery_. The ratio of CD31_inside_ and CD31_periphery_ was calculated using the formula: ratio = (total CD31_inside_)/(total CD31_periphery_).

Blinded Ki67 IF images were quantified by two unbiased observers. Cells exhibiting a clear Ki67^+^ and DAPI^+^ staining were counted as proliferative. The proliferation rate was calculated using the formula: proliferation rate = (Ki67^+^/DAPI^+^ cells in pseudo-islet)/(DAPI^+^ cells in pseudo-islet).

### Statistical analysis

Statistical analysis was performed using Origin 2018 (OriginLab, Northampton, MA). Results shown throughout the entire article are means ± standard deviations. All data sets were tested for normal distribution with Kolmogorov–Smirnov test. Outliers were identified with Grubb's test. Significance of the CD31/insulin ratio was analyzed using one-way analysis of variance (ANOVA) (*n* ≥ 6). The statistical significances of the TUNEL assay were analyzed with Student's *t*-test and one-way ANOVA, respectively (*n* = 10). GSIS data were statistically analyzed using two-way ANOVA with Tukey's multiple comparison for the effect of aggregation process and spatial distribution (*n* ≥ 3). The GSIS index from Krebs 1 to glucose and from glucose to Krebs 2 was statistically analyzed by one-way ANOVA with Tukey's multiple comparison (*n* ≥ 3). Pseudo-quantification of E-cadherin was assessed using one-way ANOVA with Tukey's multiple comparison for the effect of spatial distribution (*n* = 20). The β-cell and HUVEC pseudo-islets formed by spontaneous aggregation or magnetic levitation were statistically analyzed using one-way ANOVA with Tukey's multiple comparison for the effect of time on pseudo-islet size (*n* = 10). GSIS data of functionality assessment were statistically analyzed by two-way ANOVA with Tukey's multiple comparison (*n* = 10). The corresponding GSIS index from Krebs 1 to glucose and from glucose to Krebs 2 was statistically analyzed by one-way ANOVA with Tukey's multiple comparison (*n* = 10). Area comparison and total CD31^+^ content were statistically analyzed using Student's *t*-test (*n* = 10). Relative cell number during excision was statistically evaluated using one-way ANOVA (*n* = 7). The significance of proliferation rate was analyzed using Student's *t*-test (*n* = 10). Statistical significance was defined at *p* < 0.05.

## Results

### Magnetic levitation facilitates controlled aggregation of heterotypic pseudo-islets and HUVEC integration

**FIG. 3.**
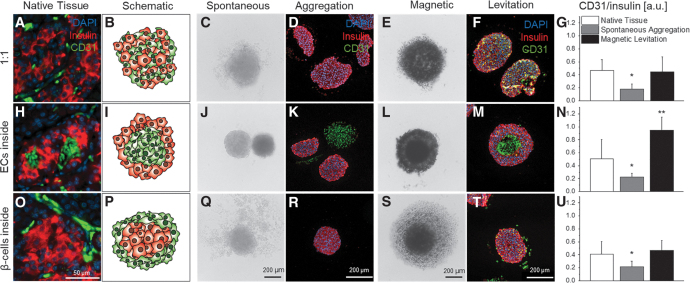
Spatial distribution of β-cells and ECs in native pancreas tissue, representative BF and IF staining images of *in vitro*-formed heterotypic pseudo-islets with spontaneous aggregation or magnetic levitation as well as CD31/insulin analysis. Native tissue staining for CD31 as EC marker and insulin as β-cell marker revealed different spatial distributions of ECs and β-cells within the human pancreas **(A, H, O)**. Spatial distributions are shown in the schematic depiction for 1:1 **(B)**, ECs inside **(I)**, and β-cells inside **(P)** (ECs in *green*, β-cells in *red*). BF images of heterotypic pseudo-islets in culture and IF images after histological preparation of heterotypic pseudo-islets formed by spontaneous aggregation (**C, D, J, K, Q, R**) showed no interaction and disassemble of HUVECs independent from the spatial distribution. BF and IF images of heterotypic pseudo-islets formed with magnetic levitation (**E, F, L, M, S, T**) showed improved interaction and controlled aggregation. HUVEC integration was significantly increased with magnetic levitation and comparable with native levels (**G, N, U**). Student's *t*-test, *n* = 6, *p < 0.01, **p < 0.001. BF, bright field; DAPI, 4′,6-diamidino-2-phenylindole; IF, immunofluorescence.

In native pancreatic tissue (Fig. 3A, H, O), we identified three major naturally occurring spatial distributions of β-cells and ECs: (1) the heterogeneous distribution of both cell types (“1:1,” Fig. 3A), (2) ECs surrounded by β-cells (“ECs inside,” Fig. 3H), and (3) β-cells surrounded by ECs (“β-cells inside,” Fig. 3O). Based on these findings, schematics were developed to model the distributions in heterotypic pseudo-islets that needed to be evaluated (Fig. 3B, I, P).

**FIG. 4.**
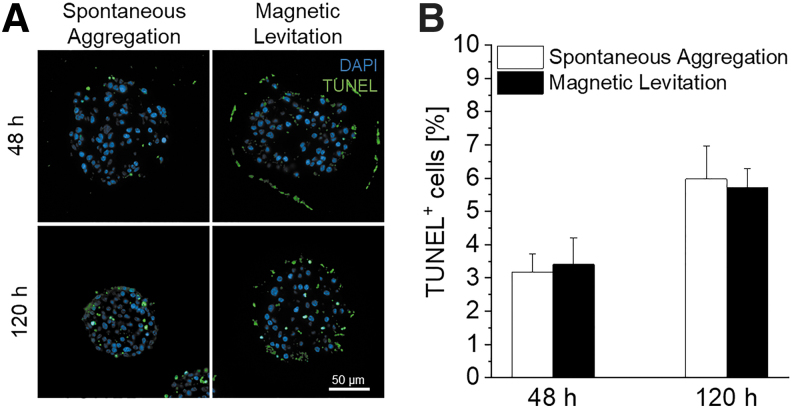
Evaluation of influence of NanoShuttle™-PL treatment on DNA fragmentation of cells, indicating the final stage of apoptosis and necrosis. **(A)** TUNEL staining of pseudo-islets formed with spontaneous aggregation and magnetic levitation after 48 and 120 h. **(B)** Quantification of TUNEL^+^ cells per DAPI. Student's *t*-test, *n* = 10. TUNEL, terminal deoxynucleotidyl transferase-mediated nick-end labeling.

To mimic the three distribution patterns seen in native pancreatic tissue, and in addition to spontaneous aggregation, we utilized magnetic levitation for a controlled cellular movement and aggregation by employing external magnetic fields. To identify a potential impact of the NanoShuttle-PL treatment on cell survival, TUNEL assays were performed to evaluate DNA fragmentation within cells as an indication of the last step of cell apoptosis or necrosis.^34,35^ TUNEL analysis revealed no significant differences in the number of cells exhibiting DNA fragmentation within the β-cell-containing pseudo-islet cultures after 48 h (3.19 ± 0.53% spontaneous aggregation vs. 3.41 ± 0.80% magnetic levitation, *p* = 0.922) or 120 h (5.97 ± 1.01% spontaneous aggregation vs. 5.71 ± 0.58% magnetic levitation, *p* = 0.887) (Fig. 4). Furthermore, glucose response of pseudo-islets after 120 h was maintained after NanoShuttle-treatment (Supplementary Fig. S2).

**FIG. 5.**
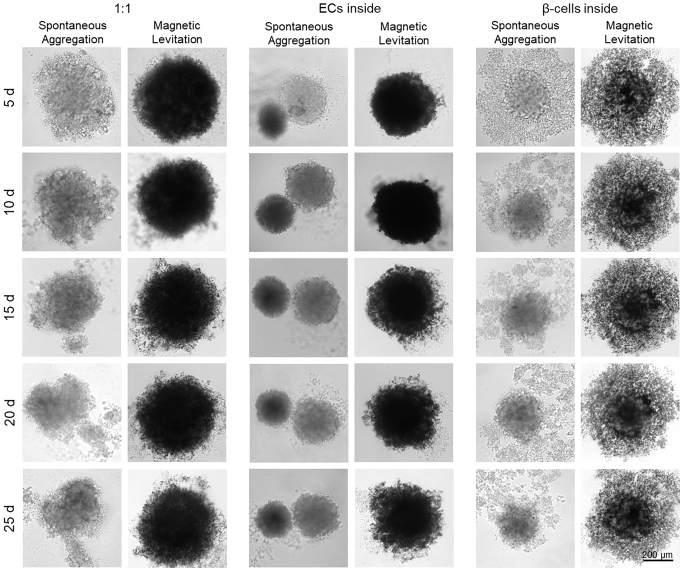
Representative BF images of long-term stability tests of heterotypic pseudo-islets using spontaneous aggregation and magnetic levitation for up to 25 days with minor disassembly occurring after day 15. Heterotypic pseudo-islets formed with magnetic levitation were stable for up to 25 days independent from spatial distribution. Pseudo-islets formed with spontaneous aggregation show disaggregation starting at day 10 for “1:1” pseudo-islets, at day 5 for “ECs inside” pseudo-islets and at day 10 for “β-cells inside” pseudo-islets.

Spontaneous aggregation led in all three distribution types (“1:1,” “ECs inside” and “β-cells inside”) to the disassembly of HUVECs from the β-cell-composed pseudo-islets (Fig. 3C, D, J, K, Q, R). IF staining revealed that almost no CD31^+^ HUVECs were present within the insulin-producing β-cell-composed pseudo-islets (Fig. 3C, J, Q).^36,37^ In contrast, magnetic levitation resulted in the formation of intact heterotypic pseudo-islets with a promoted HUVEC integration (Fig. 3E, F, L, M, S, T). These heterotypic pseudo-islets were stable for up to 25 days (Fig. 5). IF staining revealed that CD31^+^ HUVECs were heterogeneously distributed throughout the “1:1” pseudo-islets (Fig. 3F). The “ECs inside” condition produced pseudo-islets that showed a distinguishable CD31^+^ HUVEC center (Fig. 3M). IF staining of the pseudo-islets formed based on the “β-cells inside” distribution pattern showed predominantly CD31^+^ HUVECs in the periphery of the constructs (Fig. 3T; Supplementary Fig. S3).

According to these observations, quantification showed a statistically significant increase in CD31^+^ HUVECs in all pseudo-islet distribution types formed by magnetic levitation when compared with pseudo-islets formed by spontaneous aggregation (“1:1”: 0.45 ± 0.23 vs. 0.18 ± 0.07, **p < 0.01, Fig. 3G; “ECs inside,” 0.95 ± 0.20 vs. 0.23 ± 0.05, ***p < 0.001, Fig. 3N; “β-cells inside,” 0.47 ± 0.15 vs. 0.22 ± 0.08, **p < 0.01, Fig. 3U).

### Spatial distribution of heterotypic pseudo-islet cultures significantly influences insulin secretion upon glucose stimulation

**FIG. 6.**
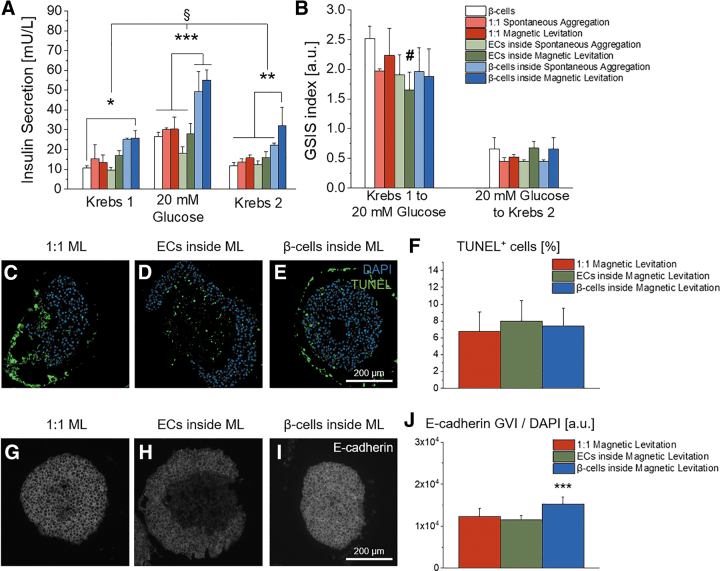
Functionality assessment of heterotypic pseudo-islets formed with spontaneous aggregation and magnetic levitation. **(A)** “β-cells inside” secreted significantly more insulin upon glucose challenge. In addition, “β-cells inside” formed with magnetic levitation had significantly increased levels of basal insulin secretion; one-way ANOVA, *n* ≥ 3. **(B)** The GSIS index for “β-cells inside” did not differ significantly showing comparable stimulation properties although basal insulin secretion is increased; one-way ANOVA, *n* ≥ 3. **(C–E)** Representative images of TUNEL staining of heterotypic spheroids with the three different spatial distributions formed with magnetic levitation. **(F)** Quantification of TUNEL^+^ cells per DAPI. One-way ANOVA, *n* = 10. **(G–I)** Representative images for GVI analysis of IF staining for E-cadherin and **(J)** pseudo-quantification of heterotypic pseudo-islets formed by magnetic levitation; one-way ANOVA, *n* = 20. ^§^*p* < 0.001, ^#^*p* < 0.01, *p < 0.05, **p < 0.01 ***p < 0.001. GVI, grey value intensities.

We assessed the functionality of the pseudo-islet cultures by measuring the insulin secretion using serial GSIS assays (Fig. 6A, B). We identified that pseudo-islets composed of β-cells inside and HUVECs outside secreted significantly higher amounts of insulin upon glucose stimulation, independently of the aggregation method when compared with all other cell compositions or the control group, which is pseudo-islets composed of β-cells alone formed by spontaneous aggregation (“β-cells inside” spontaneous aggregation: 49.37 ± 10 mU/L vs. “β-cells inside”: magnetic levitation 55.09 ± 5.01 mU/L, ***p < 0.001, Fig. 6A). Interestingly, the pseudo-islets composed of β-cells inside and HUVECs outside formed by magnetic levitation produced significantly higher levels of insulin in the initial basal state after Krebs 1 medium treatment when compared with the control group (25.85 ± 3.80 mU/L vs. 10.59 ± 1.29 mU/L, *p < 0.05, Fig. 6A). The “β-cells inside” condition formed by magnetic levitation also secreted significantly higher levels of insulin in the second basal state, after Krebs 2 medium treatment, when compared with any other condition and the control group (**p < 0.01, Fig. 6A). Importantly, no relevant statistically significant differences in the GSIS indices calculated from the serial GSIS were observed (Fig. 6B), which indicates that the stimulable nature of β-cells is not altered in the “β-cells inside” distribution group, although the basal insulin levels are increased. Importantly, the observed differences in glucose response were not attributable to cell death, since TUNEL analyses of the three spatial distributions formed by magnetic levitation did not exhibit any significant differences in DNA fragmentation (“1:1”: 6.76 ± 2.32%, “ECs inside”: 7.99 ± 2.44%, “β-cells inside”: 7.40 ± 2.10%, Fig. 6C–F).

In summary, we identified that heterotypic pseudo-islets with β-cells inside and HUVECs outside formed by magnetic levitation exhibited the highest insulin secretory function in every condition without a loss in stimulation capabilities from basal to high glucose and back to the basal state.

### β-Cells surrounded by HUVECs express significantly more E-cadherin

E-cadherin is a cell surface adhesion protein that is associated with cell–cell interactions and has been proposed to promote insulin secretion through intra-islet communications.^38–40^ In this study, we investigated the expression of E-cadherin in the heterotypic pseudo-islets formed by magnetic levitation (Fig. 6G–J). We identified that E-cadherin was distributed evenly throughout the “1:1” and “β-cells inside” heterotypic pseudo-islets (Fig. 6G, I). In contrast, pseudo-islets composed of ECs inside and β-cells outside did not express E-cadherin in the center (Fig. 6H). In general, we saw that only β-cells expressed E-cadherin. Interestingly, β-cells of the “β-cells inside” condition expressed significantly higher levels of E-cadherin per cell when compared with the other conditions (“β-cells inside”: 75.75 ± 15.15 compared with “1:1”: 55.17 ± 5.93 and “ECs inside”: 47.02 ± 6.39; *p* < 0.001, Fig. 6J).

## Discussion

Pancreatic β-cells, which are naturally surrounded by a major capillary network that provides proper nutrition, oxygenation, cell–ECM, and cell–cell interactions, suffer from loss of this microenvironment after transplantation.^41^ Recreating the pancreatic niche by the combination of ECs and pancreatic β-cells may contribute to graft survival *in vitro.*^42^ Apart from improved graft survival^13,22^ and prevascularization,^43^ the coculture of β-cells with ECs provides beneficial effects on β-cell functionality.^10,18,27^ However, these stimulatory capabilities of ECs on β-cells have not been shown to exist in a human-only cell line-based *in vitro* model. In addition, the influence of the spatial distribution in such human heterotypic cell aggregates has not been clarified. Yet, both are requirements that need to be met to understand factors contributing to regulatory mechanisms in the crosstalk of β-cells and ECs in the human body.^44^

In this study, we identified three major spatial distributions of β-cells and ECs in native human pancreatic tissue: (1) the even distribution of both cell types, (2) ECs surrounded by β-cells, and (3) β-cells surrounded by ECs. We introduced an abstract classification (1–3) of the complex anatomy to recreate an *in vitro* system based of these naturally occurring distributions. Furthermore, investigation of the spatial distribution of the islets of Langerhans revealed that each β-cell in native islets is surrounded by at least one EC,^9,11,18^ underlining the relevance of all three selected spatial distributions. To recapitulate those three distribution patterns, “1:1,” “ECs inside” and “β-cells inside,” heterotypic pseudo-islets consisting of human β-cells and HUVECs were formed *in vitro* by spontaneous aggregation. In contrast to previous studies showing natural aggregation of HUVECs with murine MIN6 cells,^18^ in our hands, HUVECs did not interact with the human β-cells. Moreover, the HUVECs formed rather autonomous spheroids, and a formation of stable pseudo-islets with defined structural features was not possible. One limitation could be that HUVECs are not an ideal cell source to mimic vascular ECs in combination with the human β-cell line EndoC-βH3, although HUVECs have been widely used and established to model ECs behavior.^5,18,25,27,43,45–48^ Dissimilarities upon cytotoxic reagents or in cytoarchitecture of islets have been previously described, underlining major differences between human and rodent pancreatic islets, which limits the possibility to extrapolate from results obtained in rodent systems to the human ones.^29,49^

Since β-cells rely on an highly specialized environment,^15^ a human cell line-based *in vitro* test system might require particularly specialized ECs to properly model the interaction between β-cells and ECs, such as human pancreatic microvascular ECs or ductal epithelial cells (DECs). DECs, for instance, play an important role in pancreatic morphogenesis^50^ as well as during initial islet differentiation.^51^ Furthermore, it has been shown that insulin-producing β-cells can be derived from DECs^52,53^ since both cell types originate from foregut endodermal progenitors,^51^ hinting toward comparable specialized capabilities and properties. In addition, different groups found that DECs increase the viability and proliferation of mature β-cells by secretion of islet growth and regulatory factors such as insulin-like growth factor 2, thus naturally contributing to the maintenance of the β-cell population in the pancreas *in vivo.*^50,54^

To overcome the interactional problem between HUVECs and human β-cells, we used magnetic levitation to form heterotypic pseudo-islets. This technique uses positively charged poly-l-lysine amino chains to attach gold and iron oxide nanoparticles to the cell surface, which in turn enables a controlled cell aggregation by application of external magnetic fields.^55^ By utilizing magnetic levitation, we created pseudo-islets in a controlled manner with defined internal architectures and improved HUVEC integration. Magnetic levitation helps, therefore, to overcome the inefficient incorporation of different cell types in heterotypic spheroids as seen when using spontaneous aggregation like in this study and as it was described previously by Kusamori *et al.*^14^ This method is also improving the reproducibility of mixed heterotypic spheroids and addresses the limitation of conventional methods to control the size of multicellular spheroids.^14,56^

In our study, pseudo-islets were derived using 5000 cells/cell type per spheroid with magnetic levitation. TUNEL assays showed that the cell viability within the pseudo-islets was not impacted by the method. Structural and distributional differences can be easily monitored in bigger heterotypic spheroids, facilitating also the quantification of cell–cell and cell–matrix integrations. With the successful establishment of the human cell line-based *in vitro* model, further studies can now be conducted with smaller pseudo-islet sizes ranging in the native pancreatic islet size of ∼150 μm, improving both functionality and survival of pseudo-islets.^57–60^ A limitation of the current setup are the varying culture times of the three different coculture pseudo-islet types, which originated from external constraints: (1) pseudo-islets need 48 h to form stable aggregates, independent from cell type and culture method, and (2) the mandatory culture time for the β-cells of 5 days before GSIS according to manufacturer's protocol (Fig. 2).

We were further interested in the question of how multicellular spatial distributions, as seen in native human pancreas, may impact insulin secretion upon glucose stimulation within the *in vitro*-engineered pseudo-islets. The spatial influence of ECs during pancreatic development has been described before, showing that the dorsal aorta initiates the endocrine differentiation, which can be monitored by an increased insulin expression and by the formation of pancreatic bud-like structures that form from the endoderm.^61^ When Lammert *et al.* removed the dorsal aorta in their study, no insulin gene expression was seen.^61^ During development, the spatial distribution of ECs and blood vessels adapt as the pancreas forms from an adjacent distribution of ECs and endoderm to a dense meshwork of capillaries within the fully developed pancreas,^62^ supporting the important role of ECs for endocrine functionality. In this study, we identified that only constructs with β-cells inside and HUVECs outside achieved a significant insulin secretion stimulation upon glucose challenge. Although both aggregation procedures, spontaneous aggregation and magnetic levitation, showed comparable amounts of secreted insulin, a significant stimulation in basal insulin secretion was solely identified for the “β-cells inside” constructs formed by magnetic levitation (Fig. 6A). Importantly, no significant alteration in any of both GSIS indices could be observed. Loss in GSIS index would be an epiphenomenon of impaired intracellular feedback loops and β-cell mis-sensing of extracellular levels of glucose and insulin,^63^ undermining the validity of this *in vitro* test system.

In our hands, an increase in β-cell insulin secretion could only be achieved when HUVECs were added to preformed β-cell-composed pseudo-islets. Thus, spatial distribution is critical to enhance β-cell functionality. The “β-cell inside” distribution pattern has also been used by Ilieva *et al.*,^50^ Ferrer *et al.*,^53^ and Murray *et al.*^54^ to stimulate insulin secretion, although not by design, since isolated pancreatic islets had been used in their setup, which are already solidly formed. In contrast, Kusamori *et al.*^14^ reported spontaneous formation of the “ECs inside” distribution when adding murine ECs, fibroblasts and β-cells simultaneously to form heterotypic spheroids, which also led to an increased insulin secretion. This might be explained by optimal cell–cell interactions in this intraspecific rodent model consistent of mouse ECs, mouse fibroblasts, and mouse β-cells.^14^ Although nearly no HUVECs were integrated when using spontaneous aggregation, also those conditions were able to be stimulated by coculture. This phenomenon has already been described by groups that used EC-conditioned media to upregulate β-cell functionality by introducing factors expressed by ECs into the β-cell culture.^9,50^ Our results indicate that either the self-assembly of β-cells inside and HUVECs outside integrates a sufficient percentage of HUVECs to achieve β-cell stimulation, or that β-cells only require signalling molecules expressed by HUVECs to be stimulated.

Heterotypic pseudo-islets with β-cells inside and HUVECs outside expressed significantly higher levels of the cell–cell adhesion marker E-cadherin than in any other spatial distribution (Fig. 6G–J). E-cadherin has been shown to promote intracellular insulin secretion^38–40^ by its binding properties to β-catenin, thus controlling actin skeleton remodeling through α-catenin,^64^ which is known to regulate insulin secretion from β-cells.^65^ In addition to cell–cell adhesion, E-cadherin functions as cell proliferation mediator within the islets through the Wnt pathway, contributing to β-cell mass stability.^40,66^ Since HUVECs express only VE-cadherin and neuronal (N-) cadherin,^67^ yet no E-Cadherin (Fig. 6H), the integration of HUVECs into the pseudo-islets possibly disturbed the β-cell communication in the “1:1” and “ECs inside” distributions, whereas the “β-cells inside” constructs were formed without any HUVEC intermission. Since both cadherins can bind p120, another cell adhesion protein related to β-catenin, VE-cadherin might interfere with E-cadherin and hence interrupt the cell–cell signalling between the β-cells, resulting in stimulated insulin secretion solely in the “β-cells inside” condition.^64,68–70^ To summarize, the spatial distribution, which can be controlled by using magnetic levitation, is critical to stimulate the EndoC-βH3 cell functionality by HUVEC coculture. Only when cultured as “β-cells inside” distribution, β-cells exhibited an increased insulin secretory function, accompanied with elevated levels of E-cadherin, which is vital for cell–cell communication and insulin expression.

A pathway through which ECs might upregulate β-cell functionality is through interaction of CD31 with the integrin α_v_β_3_, since CD31 expressed by ECs is a counter-receptor of α_v_β_3._^37,71,72^ CD31 regulates angiogenesis, migration, proliferation, and cell–cell interaction by homophilic binding, but has also been shown to interact heterophilic.^71^ An interaction of CD31 with the α_v_β_3_-integrin present on the cell surface of β-cells^73^ might also promote β-cell functionality and survival. It has been described that the binding of α_v_β_3_ activates the ERK1/2-pathway, which is known to increase an intercellular insulin response.^73–75^ However, the activation of α_v_β_3_ by CD31 alone cannot completely explain the increased β-cell functionality, since heterotypic spheroids with the “ECs inside” distribution revealed the highest CD31 ratio without a significantly increased insulin secretion, implying that other pathways take place in the stimulation process of β-cells.

## Conclusion

In this study, we have established the first human cell line-based pseudo-islet *in vitro* test system to investigate coculture effects of human β-cells on human ECs. Magnetic levitation allowed the stable formation of heterotypic pseudo-islets with defined spatial distributions of β-cells and HUVECs. In addition, we showed that the stimulatory capabilities of ECs on β-cells were best utilized when a β-cell composed pseudo-islet is surrounded by an outer layer of ECs, emphasizing that the spatial distribution as well as cell–cell interactions are crucial for an increased insulin secretion and, therefore, β-cell functionality. Together, these promising results lay the foundation for upcoming study to further improve the *in vitro* test model and investigate coculture interactions of human β-cells and ECs en route to develop prevascularized transplantable islet grafts.

## Supplementary Material

Supplemental data
