# Supplementary material for: Controlled Heterotypic Pseudo-Islet Assembly of Human β-Cells and Human Umbilical Vein Endothelial Cells Using Magnetic Levitation
Source: Tissue Eng Part A. 2020 Apr 16;26(7-8):387–99. doi: 10.1089/ten.tea.2019.0158 (PMC7187983; doi:10.1089/ten.tea.2019.0158)
Supplement: Supplemental data [file Supp_FigS1-S3.pdf]

Supplementary Data

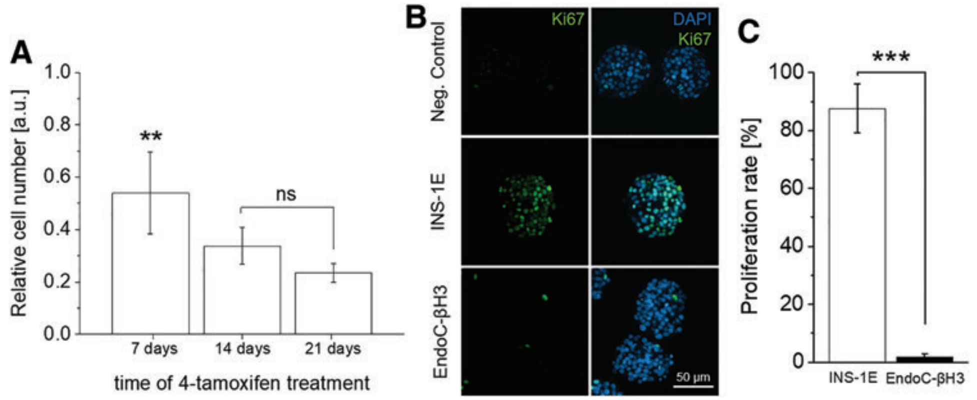

SUPPLEMENTARY FIG. S1. Demonstration of successful removal of proliferation gene of EndoC-βH3 cells by 4-tamoxifen treatment during excision. **(A)** Reduction of proliferation rate during treatment time. One-way ANOVA, $n=7$, $^{**}p<0.01$. **(B)** Ki67 staining of rat insulinoma cells (INS-1E) pseudo-islets as positive control for proliferation and EndoC-βH3 pseudo-islets. **(C)** Percentage of proliferating cells in INS-1E and EndoC-βH3 pseudo-islets. Student's *t*-test, $n=10$, $^{***}p<0.001$. ANOVA, analysis of variance; DAPI, 4′,6-diamidino-2-phenylindole; ns, not statistically significant.

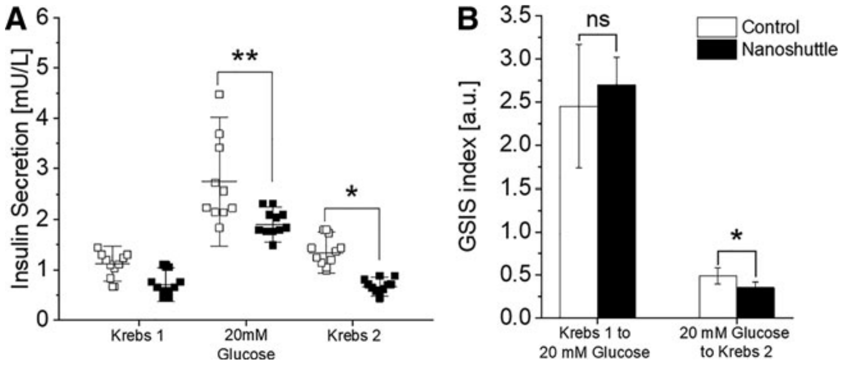

SUPPLEMENTARY FIG. S2. Functionality assessment in three-dimensional after treatment with NanoShuttle™. **(A)** Insulin secretion after serial GSIS assay of pure β-cell pseudo-islets with 1000 cells/pseudo-islets after 5 days in culture. Two-way ANOVA, $n=10$, $^{*}p<0.05$, $^{**}p<0.01$. **(B)** GSIS index of serial GSIS assay showing no relevant differences in functionality after treatment with NanoShuttle™. Student's *t*-test, $n=10$, $^{*}p<0.05$. GSIS, glucose-stimulated insulin secretion.

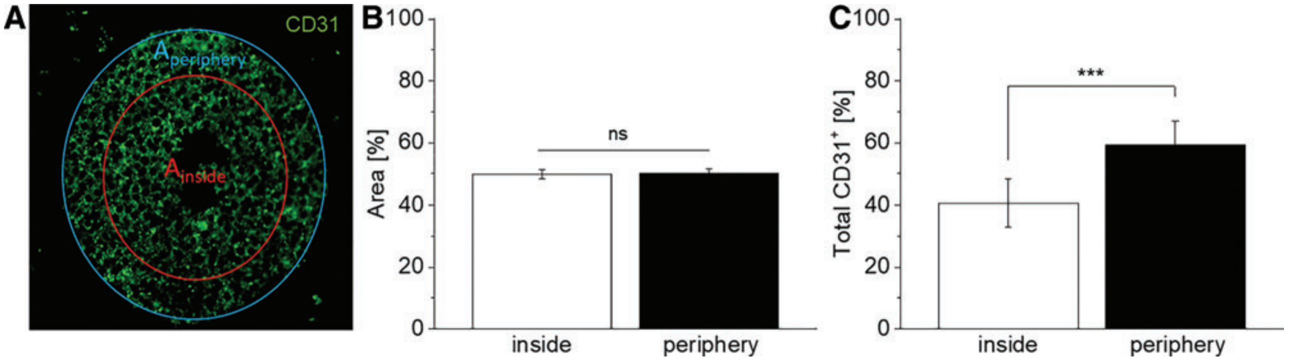

SUPPLEMENTARY FIG. S3. Analysis of the distribution of endothelial cells in ''β-cells inside'' formed with magnetic levitation. **(A)** Schematic of the area of interest that was evaluated as ''inside'' and ''periphery.'' **(B)** Area ratio of ''inside'' and ''periphery.'' Student's *t*-test, $n=10$. **(C)** Total $CD31^{+}$ staining within areas ''inside'' and ''periphery'' showing that $CD31^{+}$ cells are mainly located in the periphery of ''β-cells-inside'' formed with magnetic levitation. Student's *t*-test, $n=10$, $^{***}p<0.001$.
